# Genome size evolution at the speciation level: The cryptic species complex *Brachionus plicatilis *(Rotifera)

**DOI:** 10.1186/1471-2148-11-90

**Published:** 2011-04-07

**Authors:** Claus-Peter Stelzer, Simone Riss, Peter Stadler

**Affiliations:** 1 Austrian Academy of Sciences, Institute for Limnology, 5310-Mondsee, Austria

## Abstract

**Background:**

Studies on genome size variation in animals are rarely done at lower taxonomic levels, e.g., slightly above/below the species level. Yet, such variation might provide important clues on the tempo and mode of genome size evolution. In this study we used the flow-cytometry method to study the evolution of genome size in the rotifer *Brachionus plicatilis*, a cryptic species complex consisting of at least 14 closely related species.

**Results:**

We found an unexpectedly high variation in this species complex, with genome sizes ranging approximately seven-fold (haploid '1C' genome sizes: 0.056-0.416 pg). Most of this variation (67%) could be ascribed to the major clades of the species complex, i.e. clades that are well separated according to most species definitions. However, we also found substantial variation (32%) at lower taxonomic levels - within and among genealogical species - and, interestingly, among species pairs that are not completely reproductively isolated. In one genealogical species, called *B*. 'Austria', we found greatly enlarged genome sizes that could roughly be approximated as multiples of the genomes of its closest relatives, which suggests that whole-genome duplications have occurred early during separation of this lineage. Overall, genome size was significantly correlated to egg size and body size, even though the latter became non-significant after controlling for phylogenetic non-independence.

**Conclusions:**

Our study suggests that substantial genome size variation can build up early during speciation, potentially even among isolated populations. An alternative, but not mutually exclusive interpretation might be that reproductive isolation tends to build up unusually slow in this species complex.

## Background

Genome size, measured as the haploid nuclear DNA content (C-value), is extremely variable among eukaryotes. This variation has long puzzled biologists, because it could not be accounted by organismal complexity or the total number of genes (C-value paradox). In the last decades it has become evident that the observed genome size variation is largely caused by differences in the content of non-coding and/or repetitive DNA, such as introns, pseudogenes, or transposable elements [1,2]. Nevertheless, there are still many unanswered questions about genome size diversity, such as the actual causes driving the differences in DNA content, speed and mode of changes in genome size over population genetic and longer evolutionary time scales, or the cellular and organismal consequences of large vs. small genome size [3].

So far, most genome size comparisons in animals have been done at high taxonomic levels, e.g., between classes, families, or genera (e.g.[4-7]). Studies on genome size variation among strains or closely related species are still very scarce (but see, [8,9]). Yet, variation at these lower taxonomic levels might provide important clues on the tempo and mode of genome size evolution. Whenever two species have become separated in their evolutionary trajectories, their genome sizes might diverge neutrally, due to independently occurring processes of genome expansion and genomic deletion, unless there are factors constraining the evolution of genome size. In asexually reproducing organisms such processes might even occur on the population level. Such accumulated differences can become important, because they may contribute to genomic incompatibility, hybrid inviability, or reproductive isolation. For instance, a causal role of genome changes in speciation has been suspected for gen(om)e duplications [10,11] and transposable elements (see [12], and references therein). In this study we focus on the evolution of genome size in *Brachionus plicatilis*, a cryptic species complex consisting of at least 14 closely related species [13-15].

Cryptic species complexes have been described in many animal groups (e.g., [16-18]). In particular, small microscopic invertebrates seem to harbor large amounts of hidden genetic diversity within morphotypes that had been traditionally classified as a single species. The rotifer *B. plicatilis *is one of the most striking examples of such hidden diversity: initially described as a single species, it has experienced an enormous taxonomic inflation to currently 14-22 postulated species, based on molecular markers [14,15]. Morphological discrimination among some species of this complex is possible, however difficult, as it involves tight experimental control over environmental and developmental variation [19] or sophisticated analysis methods combined with high sample sizes [20]. Despite the morphological similarity among members of the *B. plicatilis *complex, recent studies have demonstrated extensive ecological diversification in terms of temperature or salinity preferences [21] and prezygotic and postzygotic reproductive isolation among members in this species complex [14,22,23]. In addition there are large differences in body size, with more or less continuous variation across species [14,24]. In the last decade, *B. plicatilis *has emerged as an important model organism for studies on speciation, sexual signaling [25-27], mate choice [23,28], biogeography [29,30], or selection in the wild [31,32]. *B. plicatilis *is thus also a promising future candidate for genome sequencing.

In this study we use the flow cytometry method to estimate genome size variation in the *B. plicatilis *complex. We measured the genome sizes of 33 different clones, representative of 12 cryptic species and analyze these data in a phylogenetic context. In addition, we examined body size and egg size variation in a subset of our clones and tested whether genome size is significantly correlated with any of these variables. Finally we discuss our results in the light of studies on reproductive isolation among members of the *Brachionus plicatilis *species complex.

## Results

### DNA sequencing

Blastn searches in the Genbank database using the COI (cytochrome *c *oxidase subunit I) and ITS1 (ribosomal internal transcribed spacer 1) sequences confirmed the genetic identity of our strains (Additional file 1, Table S1). The three new African isolates (Bogoria1, Nakuru1 and 2) shared 95% COI and 99% ITS1 sequence identity with isolates belonging to the *B*. 'Austria' lineage (Accession numbers: COI: AY785199, ITS1: AF387210). Our new clone OHJ4 exhibited identical sequences to previously published ones for a *B*. 'Austria' isolate (COI: AY785199, ITS1: AY772119), while those from clone OHJ1 were identical to another *B*. 'Austria' isolate (COI: AY785201, ITS1: AY772121), except for one nucleotide position in ITS1. For several clones only one of the markers was available from existing databases - either COI or ITS1 (see Additional File 1, Table S1). In these cases, we provided the missing marker sequences. Our new sequences, or sequences that were substantially longer than database sequences, have been submitted to the EMBL database with the accession numbers FR729668-FR729726.

### Phylogenetic analyses

**Figure 1:**
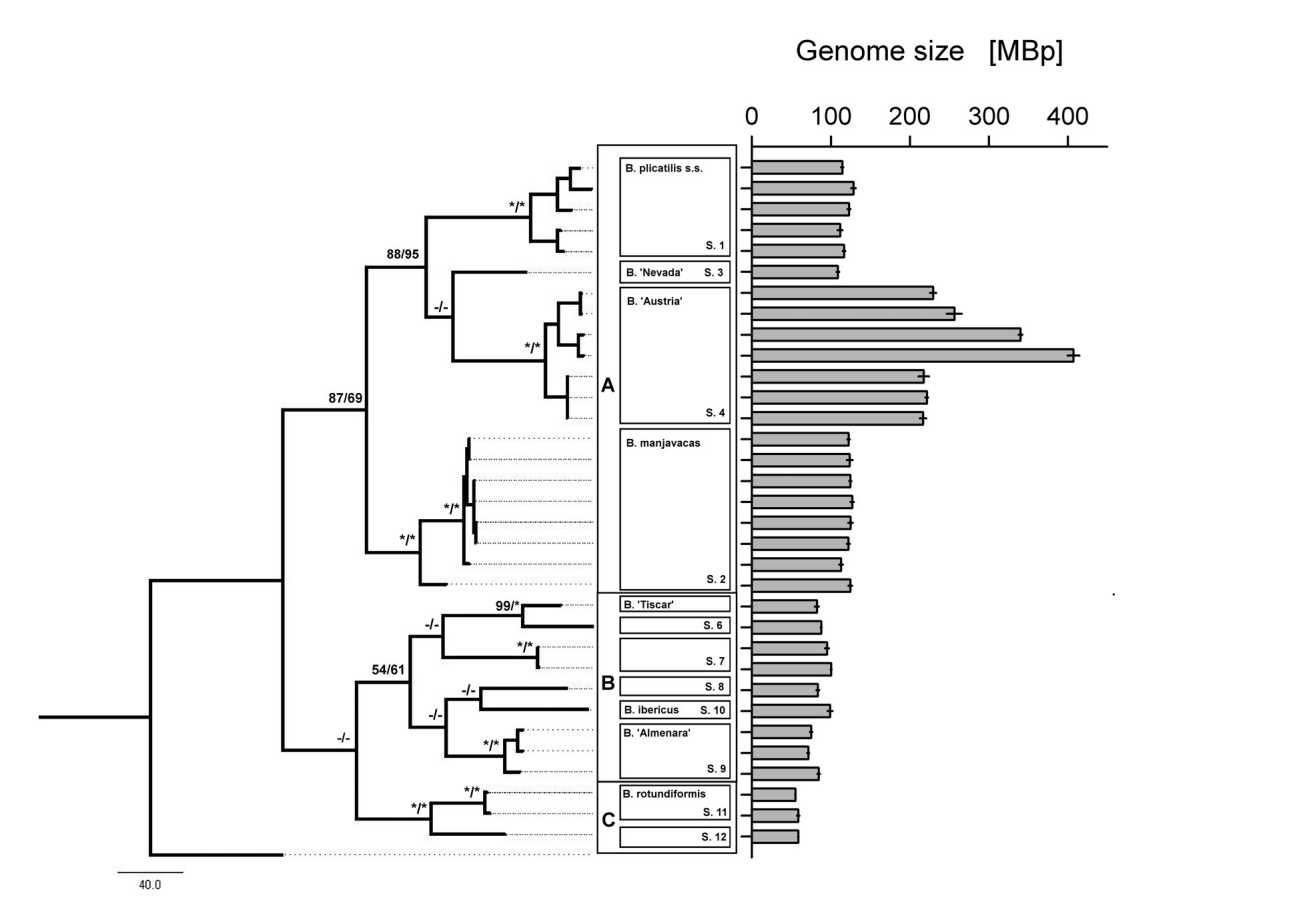
**Genome size variation in *Brachionus plicatilis *species complex**. Maximum Parsimony tree, based on combined analysis of partial mitochondrial COI and ribosomal ITS1 sequences, with *Brachionus calyciflorus *as outgroup is shown on the left. Bootstrap values for 1000 replicates for MP (1^st ^value) and NJ (2^nd ^value) are given above the branches. Asterisks indicate 100% support. Dashes indicate <50% support. Numbers of evolutionary steps are displayed as branch length. Boxes indicate the cryptic species identified by Suatoni et al (2006) and Gomez et al. (2002), as well as the three major clades (A, B, and C). Bars represent the mean haploid genome sizes of the different clones (± s.e.m.)

Both weighting schemes utilized in the MP analyses of the partial COI sequences (equal weighting; according to codon position: 1^st^: 2, 2^nd^:10, 3^rd^:1) generated the same consensus trees as the NJ analysis. Nodal support for previously described species was generally high for both phylogenetic methods (for subset 2: usually 100%, except for *B*. 'Cayman': 78 to 95% and species "S. 12" including Hawaii: 74 to 91%). In general, there were no strongly supported discrepancies in topology of the ITS1 trees resulting from the two different tree building methods. However, while all the 3 branches leading to major clades exhibited strong bootstrap support in the NJ consensus tree, the branch leading to clade B suffered from weak bootstrap support in the MP consensus trees. Employing gaps as 5th base in MP improved bootstrap support (from <50 to 68%). No disagreements among the groupings of isolates into certain clades/species between COI and ITS1 trees were found. MP analysis of the combined sequence data sets using two different weighting schemes and gap handling and NJ analysis produced consensus trees with similar topologies (Additional file 1, Figure S1), which were in accordance with previously published phylogenies [13,14]. The trees for the analysis of phylogenetically independent contrasts were constructed from the combined data set by MP method with equal weighting and treating gaps as 5^th ^base e.g. as shown in Figure 1 for subset 1.

### Genome size measurements

Haploid genome sizes ("1C") in the *B. plicatilis *complex ranged more than seven-fold, from 0.056 pg (55 Mbp) in the Adriatic2 clone, to 0.416 pg (407 Mbp) in the MNCHU008 clone of the *B*.'Austria' lineage (see Table 1 and Figure 1). Estimation of variance components showed that the largest proportion of total variance in genome size was observed among the three major clades (67%; see Table 2; clades labeled A, B, and C in Figure 1), followed by 25% of the variation that could be attributed to the species level. Variation among clones (i.e., within species) amounted only to 7%. Yet, it is evident that within species-variation in the 'Austria' lineage was higher than in other lineages with comparable phylogenetic structure (e.g., *B. plicatilis *s.s., see Figure 1 and Table 1).

**Table 1:** Genome sizes of 33 clones representing 12 different species of the *Brachionus plicatilis *species complex.

			Haploid genome size (Mbp)		
Cryptic species*		Clone	Mean	s.e.m	Replicates
*B. plicatilis s.s*.	S.1	AUBUS001	111.7	3.2	4
		AUPEA006	116.7	1.7	4
		JPNAG062	114.5	1.5	4
		L1	128.8	3.0	5
		Tokyo1	123.0	1.8	4
		**MEAN**	**118.9**		
					
*B. manjavacas*	S.2	HOY2	122.5	1.7	4
		HOY3	127.1	2.4	4
		MANL5	124.7	2.4	5
		ONT5	125.0	2.9	4
		ONT6	122.2	1.8	4
		SAL4	124.7	1.6	4
		SAL5	123.9	3.7	4
		Russia	113.1	2.5	4
		**MEAN**	**122.9**		
					
*B. 'Nevada'*	S.3	Littlefishpond2	109.1	1.7	4
					
*B. 'Austria'*	S.4	Bogoria1	217.6	6.6	6
		MNCHU008	407.0	7.4	7
		MNCHU024	340.0	2.4	6
		Nakuru1	221.5	1.7	4
		Nakuru2	216.6	4.1	4
		OHJ1	229.5	4.1	4
		OHJ4	256.3	9.6	4
		**MEAN**	**269.8**		
					
	S.6	Mortlock5	87.7	0.6	4
					
	S.7	AUYEN020	95.1	2.5	4
		Kordaclaypan56	100.4	0.8	4
		**MEAN**	**97.7**		
					
	S.8	Warrionlake37	83.5	1.9	4
					
*B. 'Almenara'*	S.9	ALM7C29	84.6	1.6	4
		Indianrocks1	75.1	1.6	5
		Lostlake1	71.4	1.4	4
		**MEAN**	**77.0**		
					
*B. 'Tiscar'*		SM28	82.4	2.6	4
					
*B. ibericus*	S.10	SM5	99.1	3.3	5
					
*B.rotundiformis*	S.11	Adriatic2	55.1	0.3	4
		HONSS	58.7	1.7	4
		**MEAN**	**56.9**		
					
	S.12	Hawaii	58.8	0.4	4

* according to Suatoni et al. (2006) and Gomez et al. (2002)

**Table 2:** Estimates of variance components of genome size.

Taxonomic level	Estimated variance component	% of total variance
Major clade	0.175	67
Species	0.066	25
Clones	0.019	7
Residual	0.003	1

Taxonomic level refers to the major clades and species designations displayed in Figure 1

Our results indicate that whole-genome duplications have occurred during the evolution of the *B*. 'Austria' lineage. Genome sizes within this lineage could be approximated roughly as multiples of 0.12 pg (117 Mbp; see Table 1 and Additional file 1, Figure S2). Interestingly, this is the average genome size of the three most closely related species (*B. plicatilis *s.s., *B*. 'Nevada', *B. manjavacas*). For instance, clones of two east African lakes (Bogoria, Nakuru) and clones isolated from Obere Halbjochlacke (OHJ1, OHJ4) had genome sizes of 0.221-0.262 pg (216 - 256 Mbp), whereas one clone isolated in Mongolia (MNCHU024) had a genome size of 0.348 pg (340 Mbp). An exception from this pattern is the MNCHU008 clone, also isolated from a Mongolian inland lake, which had a haploid genome size of 0.416 pg (407 Mbp) - clearly less than the expected 0.48 pg (467 Mbp).

### Correlations between genome size and body/egg size

**Figure 2:**
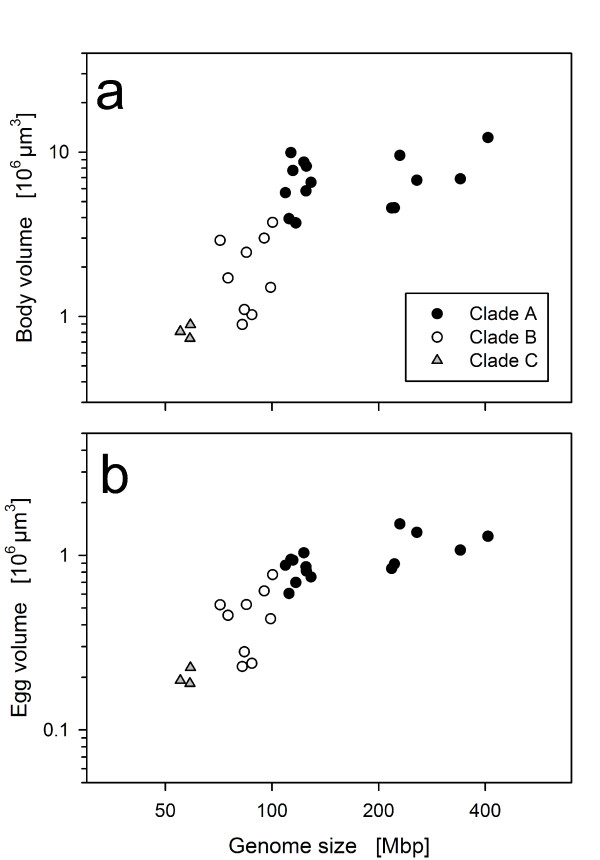
**Correlations of (a) genome size vs. body size and (b) genome size *vs***. **egg size**. Statistical analysis of these data is reported in Tables 3 and 4.

There was considerable variation in body size and egg size among the clones in our study (Figure 2). This is consistent with earlier studies [14,24], in particular, the finding that the three major phylogenetic clades showed morphological divergence into large morphotypes (clade A), medium (clade B), and small mophotypes (clade C). Interestingly this is correlated with genome size (Figure 2). Thus, we analyzed in more detail these correlations, in order to test the hypothesis that genome size might influence body or egg size variation in these eutelic organisms.

To test for phylogenetic autocorrelation, we employed the method suggested by Abouheif [33] involving 'tests for serial independence' before and after calculating PICs (phylogenetically independent contrasts) according to Felsenstein [34]. We first applied this method to our full data set of 26 clones, for which we had data on all three variables (genome size, body size, and egg size). In this full data set, phylogenetic autocorrelations could be successfully removed in the PICs of body size and egg size (Table 3). However, PICs of genome size remained significantly autocorrelated (Table 3). We assume that the reason for this were the strong genome size differences within the *B*. 'Austria' lineage, which probably represent a strong deviation from the Brownian motion model, which underlies the calculations of PICs [34]. Therefore we repeated these calculations with a dataset that excluded the *B*. 'Austria' lineage and found that phylogenetic autocorrelation could be successfully removed (Table 3). Correlation analysis on PICs calculated from this reduced dataset showed that the correlation between genome size and egg size remained significant after controlling for phylogenetic non-independence (Table 4). However the correlation between genome size and body size became non-significant (Table 4).

**Table 3:** *P*-values of the 'tests for serial independence' (Abouheif, 1999)

		ln (genome size)	ln (body size)	ln (egg size)
full dataset (26 clones)	tip data	<0.001	0.005	<0.001
	contrasts	0.006	0.295	0.449
				
reduced dataset (21 clones)	tip data	<0.001	0.005	0.003
w/o *B*. 'Austria'	contrasts	0.381	0.089	0.298

**Table 4:** Correlation analysis of genome size *vs*. body size and egg size.

		r	*P*	n
ln (genome size) *vs*. ln (body size)	raw values	0.840	<0.001	21
	PIC	0.318	0.161	20
ln (genome size) *vs*. ln (egg size)	raw values	0.847	<0.001	21
	PIC	0.521	0.016	20

Using either Pearson correlations on raw data, or phylogenetically independent contrasts (PIC).

## Discussion

The purpose of this study was to examine genome size variation in the *Brachionus plicatilis *complex, with the aim of quantifying the amounts of genome size diversification that might build up at the time scales of speciation. We found an unexpectedly high variation in this species complex, with genome sizes ranging approximately seven-fold. This variation is even higher than that observed, so far, among some rotifer species that are more distantly related and belong to different genera [35]. An analysis of the variance components showed that the largest proportions of total variance in genome size were observed at high taxonomic levels (i.e., above the species level). However, it should be kept in mind that this analysis refers the genealogical species concept. If we would apply other species concepts, some of the genealogical species would merge, resulting in a higher within-species variation (see below: *Genome size variation and speciation*)

### Whole-genome duplications

Our results suggested that whole-genome duplications have played a role in the evolution of the *B*. 'Austria' lineage. Such duplications might have been followed by gradual reductions in genome size, which could explain the lower-than-expected genome size of the Mongolian MNCHU008 clone. Interestingly, such "genome downsizing" has been frequently observed in polyploid plants, where C-values have been found to be less than expected based on the degree of ploidy [36,37]. Genome downsizing is believed to aid in the elimination of dosage effects, caused by extra DNA sequences, and to restore normal cytological and genetic behavior [37]. Nevertheless, it should be kept in mind that our conclusions about whole-genome duplications in B. 'Austria' are based solely on observations of genome size variation. Thus we cannot rule out alternative mechanistic explanations, such as a massive accumulation of retrotransposons, which might incidentally lead to similar patterns of genome size variation (e.g., [38]). Future studies are therefore needed to elucidate the exact mechanism of genome expansion in the B. 'Austria' group.

We assume that the genome size differences among clones within *B*. 'Austria' are fixed for each population and reflect divergence among populations (or, incipient species). This would be in line with other studies showing "quantum variation" of genome size among members of a taxonomic group (see [6], and references therein), even though most of these studies addressed genome size variation at higher taxonomic levels (between classes, families, or genera). An alternative explanation might be that the genome size variation observed among our 'Austria' clones is incidental, in that we might have sampled polymorphic populations consisting of clones of different ploidy and happened to sample two clones with higher ploidy in the Mongolian populations. We consider this unlikely for several reasons. First, we never found clones with genome sizes of ~0.12 pg in the *B*. 'Austria' lineage. This supports the hypothesis that at least one whole-genome duplication happened very early when *B*.'Austria' separated from the other lineages. Second, in populations from which we sampled several conspecific individuals (such as for populations of lake Bogoria and lake Nakuru) we did not find any evidence for multiple ploidy states. For the Bogoria population, we actually measured the genome sizes of several additional clones, and all these clones had genome sizes of ~0.22 pg ([35], and unpublished results). Third, substantial differences in genome size were always observed among clones that also differed in both their ITS1 and COI sequences (e.g. the two MNCHU clones), suggesting that they belong to reproductively isolated populations, rather than the same population.

### Correlates of genome size

Genome size was significantly correlated to egg size. This result is consistent with other studies showing correlations between genome size and cell size (e.g., [6]). The correlation between genome size and egg size might be expected, since rotifer egg size is determined before the first cell division. For the same reasons, it might be expected that body size is correlated to genome size, since rotifers are eutelic. However, this correlation became non-significant after we controlled for phylogenetic non-independence (Table 4). A possible explanation for the lack of significance could be developmental variance: Our *Brachionus *clones originated from a variety of habitats, from different latitudes, salinities, or water chemistry (e.g., alkaline lakes *vs*. brackish water). If they were locally adapted to these conditions, our experimental conditions (12 ppt artificial seawater, 23-24°C) might have favored somatic growth differentially.

### Genome size variation and speciation

The *Brachionus plicatilis *species complex is a good example for the difficulties in reconciling different species concepts (SC), such as the morphological SC, the biological SC, or the genealogical SC. Species boundaries in *Brachionus plicatilis *can vary considerably depending on which concept is applied [14]. For instance, while the genealogical SC suggests a clear separation of *B. manjavacas *from the other species of the clade A (see Figure 1), crossing experiments among these species often result in mating behavior [23], zygote formation, or even viable F1-offspring [14]. By contrast, the deeply diverged clades A, B, and C are well separated in terms of all species concepts, indicating that they are old [14].

The latter is consistent with our result that most of the variation in genome size (67%; see Table 2) can be ascribed to these major clades. However, we also found substantial variation at lower taxonomic levels, within and among genealogical species in each clade (32% = 25% + 7%; see Table 2). This suggests that substantial genome size variation can build up early during speciation, since many of the genealogical species identified by Suatoni et al. [14] have not yet become fully reproductively isolated. For instance, our clones "Lostlake1" and "SM5" differed 1.3-fold in genome size, yet Suatoni et al. [14] observed successful F1-offspring production in crosses between these two clones (note that "SM5" is called "Poza Sur SM" in that study). Likewise, males of the "Russian" strain (*B. manjavacas*) exhibited mating behavior towards females of the *B*. 'Austria' lineage [23], despite the fact that the genome sizes of the latter are at least 2-fold larger.

The existence of intraspecific genome size variation has been debated extensively in the past, especially among botanists [39-41]. To date, there are several case studies which unambiguously documented such intraspecific variation in plants, even though the magnitude of intraspecific variation is usually low compared to the variation observed among species (e.g., [42]). In comparison to such estimates, intraspecific variation in the rotifer *Brachionus plicatilis *seems extraordinarily high, especially if we apply the biological species concept. There are several interpretations to this pattern. First, new genome size variation might indeed be generated at very high rates in this species complex (or removed at very low rates). If this were the case, we should expect significant variability in genome size even among individuals of the same population. Unfortunately our data do not allow conclusions in this respect, since most clones derived from different populations. However future studies could address this question by examining a larger number of individuals deriving from the same population, and perhaps by examining their sexually produced offspring. This would be particularly interesting in case of the B. 'Austria' lineage. Recent studies in plants indicate that such genome size variation within populations can reach levels that are high enough to be detected by flow cytometry (reviewed by [43]). A second explanation for the high intraspecific variation in *B. plicatilis *might be that the formation of reproductive barriers in this species complex proceeds unusually slow. This is actually indicated by a general observation in the *B. plicatilis *complex: genealogical species tend to merge if the biological species concept is applied [14]. Nevertheless, it should also be kept in mind that successful *experimental *crosses between lineages (such as the "Lostlake1" and "SM5" clones, see above) do not necessarily mean that the resulting offspring would have high fitness under *natural *conditions, hence, the term 'reproductive barrier' itself is difficult to define. Future studies are therefore needed to estimate the fitness consequences of hybridization between lineages with very distinct genome sizes. Such experiments would ideally include backcrosses and fitness assays under natural conditions.

## Conclusion

In this study we observed substantial amounts of genome size variation at or slightly above the species level. This suggests that genome size variation can build up early during speciation, and that some of this variation may even accumulate among subdivided populations. It remains to be investigated to which extend such genome size variation might directly contribute to speciation.

## Methods

### Sources of strains and cultivation methods

In total, we analyzed 33 rotifer clones representing various strains of the *B. plicatilis *complex (see Additional file 1, Table S1). The majority of these clones were provided as resting eggs by several colleagues worldwide (see acknowledgments) and had been used in previous studies [13,14,23] providing information on potential species, phylogenetic relationships and cross-mating success. Further, we isolated five new rotifer clones from water samples of a small lake called Obere Halbjochlacke (Austria), and from sediment samples of two East African lakes, Lake Bogoria and Lake Nakuru. The two Mongolian clones (MNCHU008, MNCHU024) were originally collected by Christian Jersabek [44]. Clonal cultures were established from single asexual females. All culture work and experiments were done at a temperature of 23-24°C. Rotifers were grown in a modified F/2 medium at 12 ppt salinity and fed with the algae *Tetraselmis suecica *at *ad libitum *concentrations (500-1000 cells μl^-1^).

### Flow cytometry

To obtain large amounts of biomass, rotifers were cultured in 1L glass bottles, aerated with sterile air through a glass tube. The cultures were initiated with 50-100 females and were grown for 7-10 days until they reached population densities of 10-100 individuals per mL. Rotifer biomass was harvested with 30 μm sieves, resuspended in sterile culture medium and starved for 16 h. After two additional washes with sterile culture medium, cleaned rotifer biomass was resuspended in 10 mL Stock solution (3.4 mM Trisodium citrate dihydrate, Nonidet P40 at 0.1% v/v, 1.5 mM Sperminetetrahydrochloride, 0.5 mM Trishydroxymethyl-aminomethane, pH 7.6), concentrated by centrifugation (1 min at 1000 g) and buffer was removed to 0.3-1 mL (depending on the amount of initial biomass). This procedure typically resulted in harvests of 5-20 μl concentrated biomass. Rotifer biomass was ground on ice with 50 strokes in a 1 mL Dounce tissue homogenizer, to free individual nuclei. Large debris was removed by filtration through a 35 μm mesh nylon sieve. For staining of nuclei and flow-cytometric analysis we applied the method described in Stelzer et al. [45]. Briefly, 100 μl of the homogenized cell suspension was digested by addition of 450 μl of 0.003% Trypsin (dissolved in stock solution) for 10 min at room temperature. To prevent further degradation, 0.05% trypsin inhibitor was added (this solution also included 0.01% RNAse A) and the samples were incubated for another 10 min. Finally, samples were stained with propidium iodide at a concentration of 50 μg/mL. Stained samples were kept for 2-3 h on ice in the dark. Flow cytometric analysis was performed in a FacsCalibur flow cytometer (BD Biosciences) with an excitation wavelength of 488 nm and propidium iodide emission was measured in the FL2-A channels according to the manufacturer's instructions. As internal standard of known genome size we used the fruit fly, *Drosophila melanogaster *(strain ISO-1, nuclear DNA content: 0.35 pg[46]). Ten female *Drosophila *heads were homogenized in 0.5 ml Stock solution with 15 strokes in the Dounce tissue homogenizer, and 100 μl of this homogenate was co-prepared with the rotifer samples and stained in exactly the same way. Rotifer samples and *Drosophila *standard were first run separately on the cytometer, to identify the position of the peaks and to determine the approximate concentration of nuclei, and then combined into the same sample and measured again. Coefficients of variance of individual peaks were 5.5%, on average, for rotifers (down to 2.62%), and 3.9% for *Drosophila*, (down to 2.36%). Each sample was analyzed until a pre-specified number of 7,000 events (i.e., particles registered by the fluorescence detectors) were reached, typically at a rate ~30 events per second. When rotifer samples were measured in combination with the internal *Drosophila *standard, the number of counted events was increased to 15,000. In total, we analyzed 140 biomass preparations (= biological replicates) of 33 different clones. At least four replicates were prepared from each rotifer clone. To avoid bias due to temporal fluctuations in flow cytometer performance, each replicate was measured on a different day (sometimes with several weeks between two measurements). Conversion from pictograms DNA to base pairs were made with the factor: 1 pg = 978 Mbp [47].

### DNA sequencing and phylogeny

DNA was extracted from 50 to 300 μl of frozen rotifer biomass using the DNeasy Blood & Tissue kit (Qiagen). Biomass was prepared in the same way as described above for flow cytometry, except that the starved and cleaned rotifers were fixed in 70% ethanol, instead of the stock solution, and stored at -20°C. A 712-bp region of the mitochondrial cytochrome *c *oxidase subunit I (COI) was amplified and sequenced using the primers LCO1490 and HCO2198 [48]. Further, a ca. 592 to 611-bp segment containing the ribosomal internal transcribed spacer 1 (ITS1) was amplified using the primers III and VIII [49]. PCR reactions were carried out in 20 μl volumes using HotStarTaq Plus DNA Polymerase (Qiagen). Cycling parameters were: one cycle: 95°C for 5 min; 5 cycles: 94°C for 40 sec, 50°C for 40 sec, 72°c for 1 min; 35 cycles: 94°C for 40 sec, 51°C for 40 sec, 72°C for 1 min; 72°C for 10 min. Purified PCR products were sequenced by Macrogen Co. Ltd. (Seoul, South Korea). We obtained sequences for all clones of our study, even for those clones for which we already had prior sequence information, to provide an independent confirmation of their clonal identity.

A 603-bp region of COI was aligned manually. After testing a variety of different weighting levels and gap extension penalties in ClustalX, the non-coding ITS1 sequences (size range: 314 to 331 bp) were first aligned using the default options and then polymorphic sites were further manually adjusted in Bioedit. Sequences from the freshwater rotifer *Brachionus calyciflorus *were used as outgroup (COI: AF387296, ITS1: AF387243). The dataset was divided into 4 subsets containing: (1) all 33 clones used in this study (2) set 1 with additional sequences, downloaded from public databases, representing *B. plicatilis *strains not covered in this study (3) only those 26 clones of our study for which genome, egg and body size were available and (4) set 3 without the *B*. 'Austria' lineage. The main purpose for these different subsets was to ensure that confinement on a certain set of clones would not fundamentally alter their inferred phylogenetic relationship.

Phylogenetic analyses were implemented in PAUP version 4.0 b10 [50] using neighbor-joining (NJ) and maximum-parsimony (MP) methods. In general, we applied similar procedures as described in precious phylogenetic studies on the *B. plicatilis *complex [13,14]. Both markers were analyzed separately and also as a combined dataset, after testing for heterogeneity between data partitions using a partition-homogeneity test with 1000 replicates in PAUP*. MP heuristic searches (100 random taxon addition replicates) were conducted with tree bisection reconnection (TBR) branch-swapping. Searches were employed both treating gaps as a fifth state and as missing data, since gaps can hold important phylogenetic information. In the separate as well as in the combined analyses, we tried equal weighting and a weighting scheme according to the different codon positions (1^st^: 2, 2^nd^:10, 3^rd^:1) for the COI region only. Nodal support was calculated using 1000 bootstrap pseudoreplicates with the same optimality criterion utilized to construct the tree. Polytomies were forced in the tree in cases were bootstrap support was below 50%.

NJ analyses included the best fitting model of nucleotide substitution found by Modeltest 3.7 employing a hierarchical likelihood ratio test [51] for each gene and the combined data set (COI: GTR+G+I; ITS1: variable e.g. TVM+G for subset2; combined data set: GTR+G+I).

### Body and egg size measurements

Body size and egg size was measured in 26 of our experimental clones. To minimize developmental and environmental variation, we established cohorts of equally aged females, which were all processed within a single experiment using the same batches of food algae and media. Experimental females were collected as young hatchlings (age <4 h) from individually cultured parental females. Experimental females were then cultured until the age of 72 h, with daily transfers to fresh medium. At this age, they were young adults and usually carried 2-3 eggs. Females and eggs were fixed in Lugol's solution and measured using inverted microscopy at 100, 200 and 400-fold magnification and computer-aided image analysis (NIS elements, Nikon). Body size measurements included three distance measurements: total length, maximum breadth, and the breadth at the anterior end (at the base of the anterior spines). Body volume was calculated from these three distance measurements, assuming that the body shape can be approximated to a general ellipsoid with total length as the polar radius and the two breath measurements as the equatorial radii. Egg volumes were calculated from length and breath measurements, assuming that egg shape can be approximated by an ellipsoid of revolution.

### Statistics

We estimated variance components of different taxonomic levels using SPSS (version 18.0) with the REML (= restricted maximum likelihood) option selected, due to the unequal sample sizes in our data set. Correlations between genome size and egg size or body size were analyzed using Pearson correlations on log-transformed data. To test for phylogenetic non-independence in these data, we applied the algorithm described by Abouheif [33], which involves 'tests for serial independence'(TFSI). First, a TFSI was conducted on the log-transformed data, to determine whether it was at all necessary to correct for phylogenetic non-independence in our data set. Second, we calculated Felsenstein's PICs (phylogenetically independent contrasts) [34] using the PDAP module of Mesquite version 2.73 [52,53]. Third, we conducted a second TFSI, to verify that phylogenetic non-independence had been successfully removed in the PICs [33].

## Authors' contributions

CPS conceived the study, designed and participated in the experiments. PS contributed the flow-cytometry analysis. SR contributed the DNA sequencing, phylogenetic analyses, and the body/egg size measurements. CPS and SR analyzed the data and drafted the manuscript. All authors read and approved the final manuscript.

## Supplementary Material

Additional file 1 **Supplementary table and figures**. Click here for file
