# Supplementary material for: Genome size evolution at the speciation level: The cryptic species complex Brachionus plicatilis (Rotifera)
Source: BMC Evol Biol. 2011 Apr 7;11:90. doi: 10.1186/1471-2148-11-90 (PMC3087684; doi:10.1186/1471-2148-11-90)
Supplement: Additional file 1 — Supplementary table and figures. [file 1471-2148-11-90-S1.PDF]

Additional file 1. Supplementary table and figures

(Stelzer, C.P., Riss, S. Stadler, P. „Genome size evolution at the speciation level: The cryptic species complex Brachionus plicatilis (Rotifera)")

Additional file 1, Table S1: Geographic origins of the *B. plicatilis* clones and COI and ITS1 accession numbers.

Clone	Geographic origin	COI	ITS1
AUBUS001	Australia	EF524543	*this study*
AUPEA006	Australia	*this study*	*this study*
JPNAG062	Japan	GQ894745	AY772094
L1	Spain	GQ894746	DQ004843
Tokyo1	Japan	AY785175	AY772095
HOY2	Spain	DQ089844	AF387211
HOY3	Spain	EF017654	AF387211
MANL5	Spain	AF387257	AF387213
ONT5	Spain	*this study*	AF387211
ONT6	Spain	*this study*	AF387211
Russia	Russia	AF387250	AF387218
SAL4	Spain	EF017654	AF387211
SAL5	Spain	DQ089844	AF387211
Littlefishpond2	USA	AY785196	AY772116
Bogoria1	Kenya	*this study*	*this study*
MNCHU008	Mongolia	*this study*	AF387210
MNCHU024	Mongolia	*this study*	AF387210
Nakuru1	Kenya	*this study*	*this study*
Nakuru2	Kenya	*this study*	*this study*
OHJ1	Austria	AY785201	AY772119

OHJ4	Austria	AY785199	AY772119
Mortlock5	Australia	AY785209	AY772129
AUYEN020	Australia	AY785210	AY772130
Kordaclaypan56	Australia	AY785210	*this study*
Warrionlake37	Australia	AY785214	AY772136
ALM7C29	Spain	GQ894742	AF387220
Indianrocks1	USA	*this study*	AY772138
Lostlake1	USA	AY785216/9*	AY772141
SM28	Spain	AF387281	AF387231
SM5	Spain	AF387271	AF387224
Adriatic2	Italy	AY785224	AY772146
HONSS	Spain	AF387293	AF387238
Hawaii	USA	*this study*	DQ004844

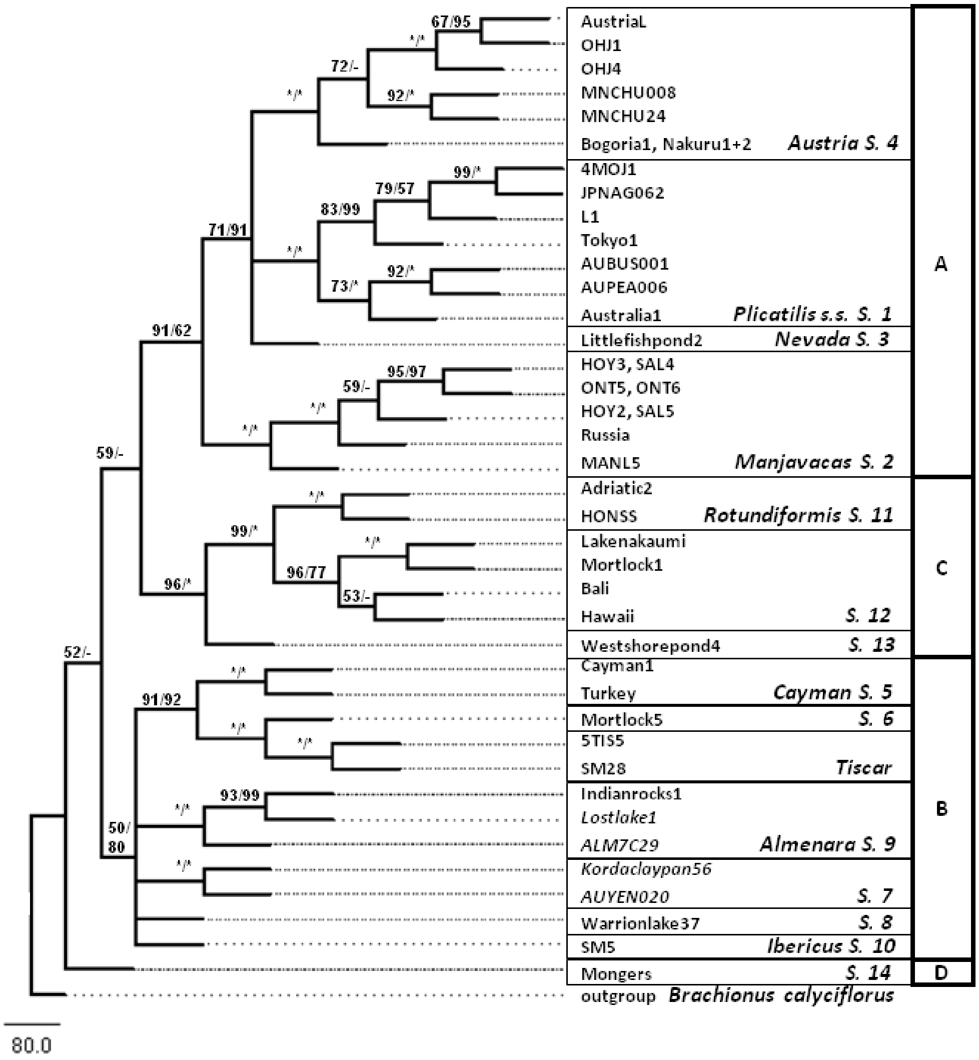

Additional file 1, Figure S1 Phylogeny of the *Brachionus plicatilis* species complex, using 44 isolates representing most of the currently recognized species in this complex (additional to our data set: lineages ‘S.5’, ‘S.13’, and ‘S.14’ from [14]). Strict consensus MP tree with *Brachionus calyciflorus* as out-group is shown based on combined analysis of partial mitochondrial COI and ribosomal ITS1 sequences. Bootstrap values for 1000 replicates for MP (1st value) and NJ (2nd value) are given above branches. Asterisks indicate 100% support. Best-fit model utilized for NJ analysis identified via Modeltest was GTR+G+I. Accession numbers of additionally downloaded COI and ITS1 sequences: 4MOJ1: AF266942, AF387203; Australia1: AF387244, AF387206; AustriaL: AF387248, AF387208; Bali: AY785226, AY772148; Lakenakaumi: AY785228, AY772150; Mortlock1: AY785227, AY772149; Westshorepond4: AY785234, AY772156; Cayman1: AF387284, AF387229; Turkey: AF387286, AF387230; 5TIS5: AF387280, AF387236; Mongers: AY785235, AY772157; only ITS1: Uganda: AY772160; Littlefishlake1: AY772158.

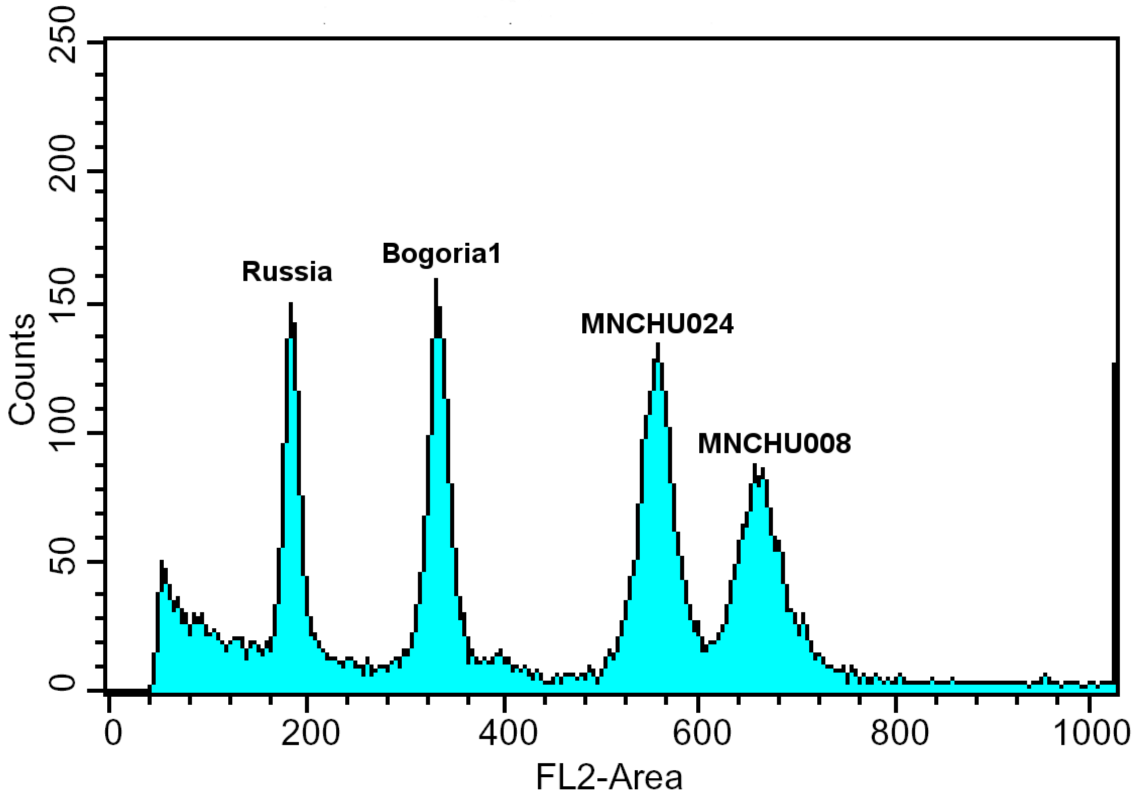

Additional file 1, Figure S2 Example of a flow cytometric run in which the samples of four different clones were analyzed together. Peaks correspond to the following clones: Russia, Bogoria1, MNCHU24, MNCHU008. Note that this analysis serves only to qualitatively illustrate genome size variation of B. 'Austria' clones in relation to a close relative ('Russia' clone). It did not include the *Drosophila* standard, which was normally loaded with each sample.
